# Present-day heat flow model of Mars

**DOI:** 10.1038/srep45629

**Published:** 2017-04-03

**Authors:** Laura M. Parro, Alberto Jiménez-Díaz, Federico Mansilla, Javier Ruiz

**Affiliations:** 1 Departamento de Geodinámica, Facultad de Ciencias Geológicas, Universidad Complutense de Madrid, 28040 Madrid, Spain

## Abstract

Until the acquisition of *in-situ* measurements, the study of the present-day heat flow of Mars must rely on indirect methods, mainly based on the relation between the thermal state of the lithosphere and its mechanical strength, or on theoretical models of internal evolution. Here, we present a first-order global model for the present-day surface heat flow for Mars, based on the radiogenic heat production of the crust and mantle, on scaling of heat flow variations arising from crustal thickness and topography variations, and on the heat flow derived from the effective elastic thickness of the lithosphere beneath the North Polar Region. Our preferred model finds heat flows varying between 14 and 25 mW m^−2^, with an average value of 19 mW m^−2^. Similar results (although about ten percent higher) are obtained if we use heat flow based on the lithospheric strength of the South Polar Region. Moreover, expressing our results in terms of the Urey ratio (the ratio between total internal heat production and total heat loss through the surface), we estimate values close to 0.7–0.75, which indicates a moderate contribution of secular cooling to the heat flow of Mars (consistent with the low heat flow values deduced from lithosphere strength), unless heat-producing elements abundances for Mars are subchondritic.

In order to have an understanding of the internal structure and the composition of Mars, as well as the thermal state of its subsurface and lithosphere, accurate knowledge of the present-day pattern of heat flow of the planet is required. Indeed, the heat flow should vary across the surface of Mars (as well as in time), depending on the abundances and distribution of the crust and mantle heat-producing elements, and on the thermal state of the planet’s interior and ability to dissipate heat. On Mars, such variations of heat flow can probably affect, among many other things, the internal geodynamics of the planet, the distribution of groundwater, ice and clathrates, and processes such as subsurface melting and fluid circulation. It is essential, therefore, in deriving global heat flow models to predict those variations throughout the planet geographically. Furthermore, the determination of average planetary heat flow provides restrictions on radioactive element content, which in turn, is important to constrain cosmochemical models and the total radioactive heat production in a planet.

Moreover, the Urey ratio (*Ur*), the ratio between the total radioactive heat production and the total surface heat loss, provides knowledge about the thermal state of a planet and whether the planet is geologically active nowadays. Thus, *Ur* constitutes an excellent means to visualize the present-day internal heat budget of the red planet, because it describes possible interior cooling or warming. Therefore, if we compared the available information on the Urey ratio throughout the history of Mars, we should be able to constraint more accurately its thermal history^1^.

Until the arrival of InSight (Interior Exploration using Seismic Investigations, Geodesy and Heat Transport), a NASA Discovery Program mission (http://solarsystem.nasa.gov/insight/overview.cfm), which includes the HP^3^ (Heat Flow and Physical Properties Probe) instrument, direct measurements of heat flow on Mars are non-existent. Foreseeably in 2018, InSight will land on the surface of Mars with the aim of determining the heat flow at the landing site in Elysium Planitia (the favored site is centered at 136°E 4°N), from the regolith thermal conductivity and the subsurface thermal gradient. In order to do this, the HP^3^ instrument, a probe (“the mole”) will penetrate up to 5 m into the martian regolith and work for one martian year^2^.

Until these *in-situ* measurements are acquired, the information currently available on past and present Martian heat flows must be deduced from indirect methods. A commonly used indirect method is based on the relation between the thermal state of lithospheric rocks and their mechanical strength, usually analyzed through the effective elastic thickness of the lithosphere (e.g., refs 3, 4, 5, 6, 7, 8) or from the depth to the brittle–ductile transition beneath large thrust faults (e.g., refs 9, 10, 11, 12). The so-obtained heat flows are valid for the time when the lithosphere was loaded or faulted, and therefore, the information deduced from regions deformed at different ages provides knowledge on the thermal evolution of Mars^8^.

Although numerous paleo-heat flow estimates have been derived using this approach, information on the present-day heat flow of this planet is limited by the difficulty in finding young structures on the surface of Mars with the polar regions currently providing the only estimates. The very limited (or non-existent) flexure caused by the north polar cap load indicates a very thick (>300 km) effective elastic thickness of the lithosphere (*T*_*e*_) on the North Polar Region (hereafter NPR)^13^. Similarly, admittance modelling of the South Polar Region (hereafter SPR) found a best fit for *T*_*e*_ of 161 km^14^. These effective elastic thicknesses can be used to estimate the present-day heat flow in those regions (refs 11,13,15; see also below), because polar caps (and the associated loading) are a recent phenomenon^16^.

On the other hand, it is recognized that large crustal thickness variations should have an expression on the surface heat flow pattern, due to the contribution of radiogenic heat production originated by the heat-producing elements (HPEs) placed in the crust^17^,^18^,^19^,^20^. The significant homogeneity of elemental abundances measured by Mars Odyssey GRS suggests that the martian crust is much less geochemically variable than the Earth’s crust^21^, which is consistent with a strong mixing by impact cratering (e.g. ref. 21), and the absence of large-scale crustal recycling, at least since the early part of the history of Mars (e.g., ref. 22), which could induce compositional differences between provinces. The influence of crustal thickness and heat production on the pattern of surface heat flow can therefore be reliably addressed, at least in a general sense.

In this work we use the currently available information on heat flow deduced from the present-day effective elastic thickness of the lithosphere, along with crustal differences in vertically integrated heat production arising from crustal thickness variations, in order to produce a preliminary global heat flow model for the present-day Mars. Previous global models were based on thermal history models^17^,^20^, whereas the present model is more empirically constrained by heat flow estimates based on lithospheric strength. Our aim is to gain understanding on the present heat flow patterns, which may be compared with our previous knowledge on the heat flow history of Mars, with thermal evolution and geochemical models, and, in the future, with the data obtained by InSight. Thus, this model should help us to advance another step in the study of the thermal state and evolution of the terrestrial planets.

## Results

### Crustal heat flows

If lateral heat transfer is neglected, the surface heat flow of Mars may be considered the sum of the heat generated in the crust and the heat flow from the mantle. In turn, the heat flow from the mantle is a consequence of the heat radioactively produced in the mantle lithosphere (or, more generally, in the stagnant lid), and of the heat coming-up from the deep interior (through mantle convection). The component of the heat flow arising from crustal radioactive heat sources is the sum of the respective contributions from all the HPEs. Global maps of elemental concentrations indicate that there is variation across the surface of Mars^21^,^23^,^24^, which is related to rock type or to extent of aqueous alteration^21^.

Previously, Hahn *et al*.^18^, obtained a crustal heat flow map based on local surface abundances of K and Th (and estimated abundances of U), considered as representative of the entire local crust, for latitudes roughly between 60°N and 60°S, because influence of volatile in higher latitudes obscures the GRS signal. However, the variations in heat production across the surface are relatively little; also, as above indicated, there are no reliable GRS data for latitudes above 60° and below −60°, where HPEs measurements are heavily affected by the presence of water molecules near the surface^18^,^24^. Thus, we here prefer to consider an average value for the crustal heat production in our model, which additionally permit modeling crustal heat flows at high latitudes (see Methods Section).

**Figure 1:**
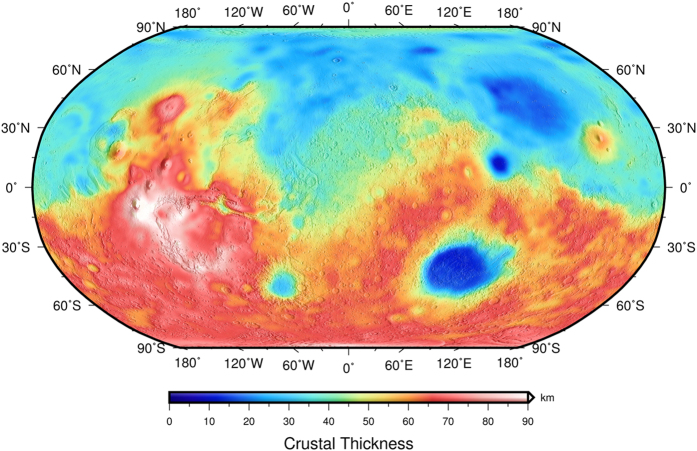
Crustal thickness model for Mars assuming a mean crustal thickness of 50 km, and crust and mantle densities of, respectively, 2900 and 3500 kg m^−3^, shown over a shaded relief map of Mars Orbiter Laser Altimeter (MOLA) topography^64^ for context.

We first derived a crustal heat flow map from crustal heat production and a crustal thickness model (for the description of the crustal thickness model see Methods Section). The crustal thickness model was derived from topography and gravity following the procedure of potential theory^25^ by assuming an average thickness of 50 km (Fig. 1). This mean crustal thickness is slightly higher than previous models^26^,^27^,^28^, but in line with geophysical and geochemical evidence^5^,^12^,^29^, and consistent with the average thickness usually used in the modeling of lithospheric strength (e.g., refs 5 and 8). In agreement with previous geophysical modeling, our crustal thickness model assumes densities of 2900 and 3500 kg m^−3^, respectively, for the crust and lithospheric mantle (see Methods Section). A crustal density of 2900 kg m^−3^ can be consistent with a basaltic crust somewhat fractured, or consistent with a basaltic crust including a felsic component. Indeed, some recent works suggest that the Martian crust could contain a substantial amount of felsic rocks^30^,^31^,^32^,^33^, although other works consider as non-solids the orbital-based evidences for a felsic component (see ref. 34). On the other hand, Baratoux *et al*.^32^ proposed a high density (>3200 kg m^−3^) for the martian basalts, although a crust so dense is incompatible with geophysical studies^32^, maybe favoring the presence of a felsic component. In any case, note that the HPEs abundances used in our model were derived from Mars Odyssey GRS observations, not assumed as a function of composition.

**Figure 2:**
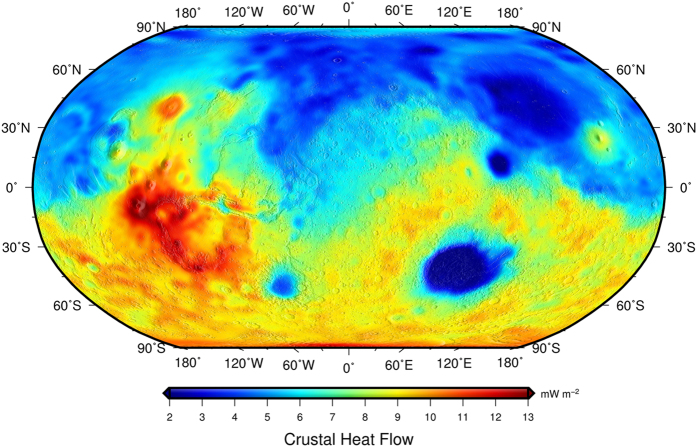
Global crustal heat flow on Mars (sampled on a 2° × 2° grid) derived from an average surface heat production based on the GRS-measured abundances of HPEs^18^,^21^,^24^, an average uniform crustal density of 2900 kg m^−3^, and our crustal thickness model.

The crustal component of heat flow varies between 1.3 and 13.5 mW m^−2^, with an average value of 7.0 mW m^−2^ (Fig. 2). The regional pattern shown by our model is similar to that observed in Hahn *et al*.^18^, albeit the absolute values are higher due to the higher crustal thicknesses of our crustal model. There is a strong correlation between the crustal thickness and crustal heat flow obtained, which implies that regional variations of crustal heat flow are strongly dependent on crustal thickness, which produces higher (lower) heat flow in zones with thickened (thinned) crust.

### Present-day heat flow model

We can scale heat flow differences across the martian surface arising from crustal thickness and topographic differences across the planet. We complete the model by assuming a constant value for the heat production in the mantle lithosphere (see Methods Section) and a constant (sub-lithospheric) heat flow from the deep interior, which would include the contribution from the convective mantle; although mantle convection should produce some regional variations of convective heat flow, we assume a constant sub-lithospheric heat flow by necessity, because we cannot specify the magnitude of these variations.

For anchoring our nominal model, we use a heat flow of 17 mW m^−2^ derived (see Methods Section) for an effective elastic thickness of the lithosphere of 300 km at the NPR, which was derived from the limited response to loading in this region^13^; lithosphere strength at the NPR currently constitutes the more reliable heat flow indicator for the present-day Mars^15^.

Although we do not adopt any specific compositional assumption for our crustal model or for crustal HPEs abundances, the composition of the crust might influence their thermal conductivity, which in turn influences the calculation of heat flows at the NPR (see ref. 8 and Methods section). The thermal conductivity used for our nominal model is 2 W m^−1^ K^−1^, a value appropriate for basalts (whose thermal conductivity is usually between 1.5 and 2.0 W m^−1^ K^−1^ 35). For the felsic terrestrial continental upper crust a thermal conductivity of 2.5 W m^−1^ K^−1^ is frequently used, although a recent revision^36^ has found that the thermal conductivity of felsic rocks in the terrestrial crust usually is not significantly higher than 2 W m^−1^ K^−1^. Because a thermal conductivity of 2 W m^−1^ K^−1^ is an upper limit for basaltic rocks, the use of this value in our nominal model is also roughly consistent with a basaltic crust including a felsic component.

**Figure 3:**
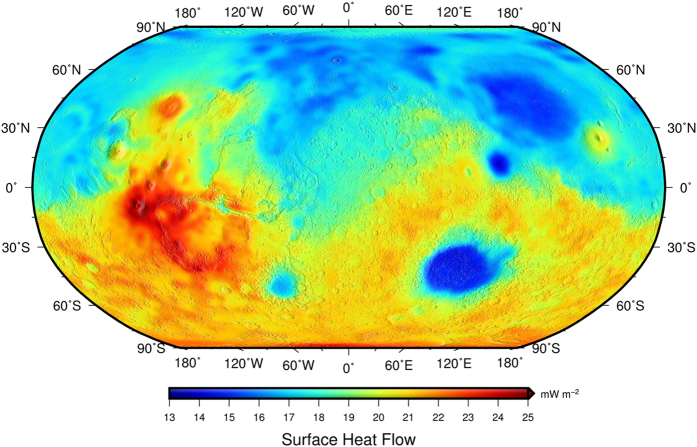
Preferred present-day surface heat flow model (the *F*_*NPR*_-based model) for Mars (sampled on a 2° × 2° grid), constructed by scaling heat flow variations related to variations in crustal thickness and topography, assuming constant mantle heat flow, and using the heat flow obtained for the NPR from the effective elastic thickness as an anchoring value.

The obtained nominal heat flow model is shown in Fig. 3. The average present-day surface heat flow is 19 mW m^−2^, and it varies between 14 and 25 mW m^−2^. The lowest values are found in the northern lowlands (e.g., Acidalia or Utopia Planitiae) and the giant impact basins (e.g., Hellas, Argyre and Isidis Planitiae) where the surface heat flow is below the global average due to relatively thin crust. Conversely, the southern highlands have a higher than average surface heat flow. The highest peaks of surface heat flow correspond to regions with very thick crust such as Tharsis Montes, Syria Planum, Alba Patera, Solis Planum, Terra Sirenum, or Thaumasia Montes.

### Present-day Urey ratio of Mars

The Urey ratio characterizes the degree of internal cooling (or heating) of a planetary body, and it can be defined as the ratio between the total radioactive heat production and the total heat loss through the surface for a given time. If *Ur* > 1 the planet interior, as an average, is heating-up; conversely, if *Ur* < 1 the planet interior is cooling-down. For Mars, if we take into account a present-day radioactive heat production equivalent to a surface heat flow of 14.3 mW m^−2^, according to the compositional model of Wänke and Dreibus^37^, and the average surface heat loss deduced from our model (19 mW m^−2^), then an Urey ratio of around 0.75 is obtained for the present-day Mars. This value is higher than the *Ur* (0.594 ± 0.024) proposed from some recent thermal evolution models^38^, but consistent with a more limited cooling deduced from lithospheric strength analysis^1^.

## Discussion

The terrestrial planets have evolved from the same primitive elements and have a similar inner structure (with differentiated core, mantle and crust), although, in addition to distance to the Sun and size, they show chemical and geological differences that make them unique within our planetary system. Major events occurred in the history of a planet (large impacts, global dynamics and environmental changes, etc.), have left signatures which define the current appearance of, and differences between, those planets. Thus, the present-day surface heat flow is very important for understanding the thermal and geologic evolution of a planetary body. Therefore, in order to improve our knowledge of the evolution of Mars, including the prospect for crustal water circulation and availability, we need to understand more fully the present thermal state of Mars, as well as the regional pattern of surface heat loss.

In absence of direct heat flow measurements for Mars, the loading of the lithosphere of the polar regions by the northern and southern polar caps (which are recent features; refs 13,16) has allowed the estimation of their respective effective elastic thickness^13^,^14^, and hence to derive estimates of the present-day heat flow^8^,^15^. In this work, we have obtained a global heat flow model (the *F*_*NPR*_-based model) for present-day Mars from (i) scaling heat flow variations related to variations in crustal thickness and topography, (ii) assuming constant mantle heat flow, and (iii) using the heat flow obtained for the NPR from the effective elastic thickness as an anchoring value. Also, for comparative and control purposes, we derive two alternative models (see Methods Section.): a model (the *F*_*SPR*_-based model) similar to the nominal model, but in this case constructed using as anchoring value the heat flow obtained for the SPR from the effective elastic thickness; and a model (the *F*_*NPRfelsic*_-based model) using as anchoring value the heat flow calculated for the NPR from a crustal thermal conductivity of 2.5 W m^−1^ K^−1^ (a very generous upper limit thermal conductivity if the crust contains a felsic component).

Our nominal (*F*_*NPR*_-based) model (Fig. 3) yields a present-day heat flow of Mars that varies between 14 and 25 mW m^−2^, with an average value of 19 mW m^−2^. Similarly, the heat flow of Mars from the *F*_*SPR*_-based model currently varies between 16 and 27 mW m^−2^, with an average value of 21 mW m^−2^. The results of both models are very similar, and therefore we propose an upper limit of 20 ± 1 mW m^−2^ for the present-day global average heat flow of Mars. In the same way, for the *F*_*NPRfelsic*_-based model we obtain an average surface heat flow of Mars of 19.6 mW m^−2^, with a variation between 14.5 and 25.7 mW m^−2^. We also obtain regional heat flow variations on Mars of, at most, ±6 mW m^−2^ with respect to the mean value. In order to verify that the results of both models are robust, we calculated the mean NPR heat flow from the *F*_*SPR*_-based model, and the mean SPR heat flow from the *F*_*NPR*_-based model, obtaining respectively 19 and 22 mW m^−2^, values within 2 mW m^−2^ of the corresponding heat flow derived from the effective elastic thickness of the lithosphere of these regions.

Geographical heat flow variations in our models are related to variations in the HPE content of the crust and mantle and, especially, to crustal thickness variations. There are other processes that could modify the surface heat flow pattern, as hydrothermal circulation^39^ or the presence of mantle plumes below the lithosphere (e.g., refs 17 and 40). There is some evidence for recent water-related geologic activity, as for example water channels flowing from Cerberus Fossae, which could be related to magma intrusion^41^, although it is not clear how this activity would affect to the background heat flow. On the other hand, the existence of mantle plumes below volcanic regions should result in a locally increased heat flow. The analysis of gravity and topography does not find evidence for substantial dynamical support for Tharsis, the largest igneous complex on Mars, and therefore for large mantle plumes active beneath this region^42^,^43^. Thus, whereas mantle heat flow variations due to mantle plumes could exist, we have ignored their possible contributions in our present model, although future evidence could require local adjustments.

Our heat flow results can also be interpreted in terms of the Urey ratio. The total radioactive heat production obtained from the bulk compositional model of Wänke and Dreibus^37^ (with chondritic HPEs abundances) can be converted to an equivalent heat flow, giving 14.3 mW m^−2^ for the present-day. Thus, for the average heat flow deduced from both the *F*_*SPR*_- and *F*_*NPR*_-based models, we obtain *Ur* of, respectively, 0.68 and 0.75; and *Ur* = 0.715 for an average heat flow of 20 mW m^−2^, the value intermediate between both models. Similarly, the Urey ratio calculated for *F*_*NPRfelsic*_-based model, *Ur* = 0.73, is consistent with our other results. These values indicate a relatively small difference between the total radioactive heat production and heat loss through the surface, and therefore a moderate contribution from secular cooling to the heat flow of Mars.

**Figure 4:**
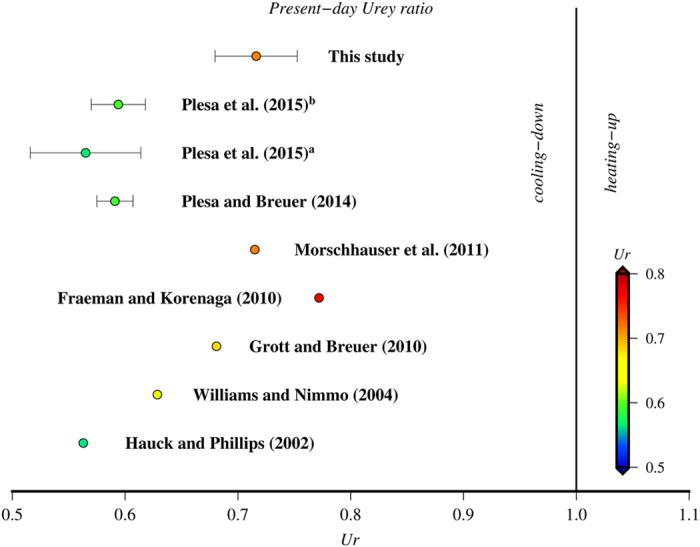
Compilation of present-day Urey ratios obtained for Mars for several convective history models compared with our results. The Urey ratio range corresponding to the present work is bounded by the results of the *F*_*NPR*_- and *F*_*SPR*_-based models, and the central dot represents the *Ur* value intermediate between the results of both models. Urey ratios from Plesa *et al*.^38^, where directly quoted by these authors (superscript indicate, respectively, 2D cylindrical and 3D spherical simulations). Urey ratios for the models by Hauck and Phillips^45^, Williams and Nimmo^46^, Grott and Breuer^17^, Fraeman and Korenaga^47^, and Morschhauser *et al*.^48^ were calculated from the present-day heat flow obtained by these models and the total heat production from the composition model of Wänke and Dreibus^37^. *Ur* = 1 separates the fields where the planet’s interior is cooling-down (*Ur* < 1) or heating-up (*Ur* > 1).

Our *Ur* results can be compared with predictions calculated by convective history models (Fig. 4). Our *Ur* values are clearly higher than those obtained from the 2D and 3D convective history models of Plesa and Breuer^44^ and Plesa *et al*.^38^, which usually vary between 0.52 and 0.62 as a consequence of higher predicted heat flows; these models would suggest therefore a substantial amount of secular interior cooling. Similarly, earlier 1D parameterized convective models predicted present-day heat flows around 23–25 mW m^−2^ (e.g., refs 45 and 46), equivalent to *Ur* ≈ 0.6 for the composition model of Wänke and Dreibus^37^; more recent and sophisticated (including melting effects on mantle viscosity and hydration) 1D convective models obtained lower heat flows, in the range between 19 and 21 mW m^−2^ (refs 17, 47 and 48), which correspond to a *Ur* values between 0.68 and 0.77 (see Fig. 4), similar to those obtained by the present work.

On the other hand, Urey ratios of ~0.6–0.7 have been proposed from the melting conditions, deduced from elemental abundances, of magmas covering some Amazonian-aged (<3 Ga) terrains^49^. However, the so-calculated mantle potential temperatures informs us on volcanic (non-average) regions, and Ruiz *et al*.^8^ show that the *Ur* values obtained by these authors are therefore lower limits. However, volcanic regions could be associated with higher than average heat flows, making any *Ur* value based on those regions a lower limit; taking this into account, these results would be consistent with our own results.

A limited influence of secular cooling through post-Noachian times is consistent with evidence for a liquid core existing at present^50^, recent abundant volcanism^51^ and limited global contraction^52^; limited secular cooling might also be consistent with high-temperature shergottite^53^,^54^, although this class of meteorites might have originated in mantle-plume settings unrepresentative of background mantle^55^. (For a review of the evidence for limited secular cooling see ref. 1). Alternatively, a low average heat flow could be explained by a sub-chondritic heat-producing elements abundances^13^; in this case, our heat flow model would give proportionally lower Urey ratios for the current Mars.

**Figure 5:**
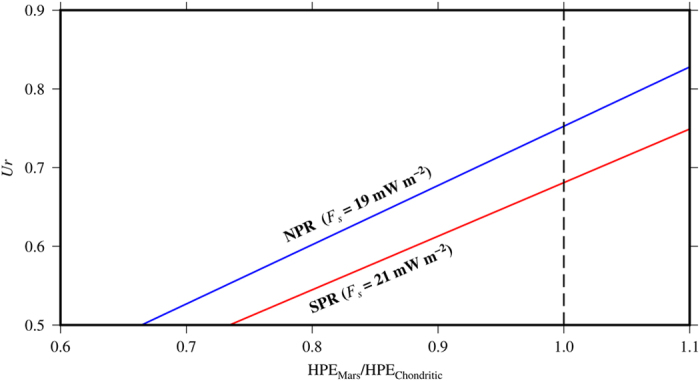
Urey ratio as a function of the HPE_Mars_/HPE_Chondritic_ ratio. The blue and red lines represent, respectively, the *Ur* values obtained from average heat flows obtained by the *F*_*NPR*_*-* and *F*_*SPR*_-based models.

Figure 5 shows the Urey ratio as a function of HPE_Mars_/HPE_Chondritic_ (where HPE_Chondritic_ represents the standard chondritic model for Mars; Wänke and Dreibus^37^). The blue and red lines represent, respectively, *Ur* values obtained from the average heat flows of the *F*_*NPR*_- and *F*_*SPR*_-based models. For example, if HPE_Mars_/HPE_Chondritic _=_ _0.9, the total radioactive heat production is equivalent to 12.8 mW m^−2^, and the corresponding value of *Ur* is 0.68 and 0.61 for, respectively, the *F*_*NPR*_- and *F*_*SPR*_-based models. Thus, for obtaining *Ur* values similar to values found by Plesa *et al*.^38^ (between 0.5 and 0.6), requires an HPE_Mars_/HPE_Chondritic_ ratio between 0.75 and 0.85, and a total radioactive heat production equivalent to ~10–12 mW m^−2^. Moreover, an Earth-like *Ur* < 0.5^36^ would require an excessively low HPE_Mars_/HPE_Chondritic_ ratio around ~0.7 or less, and we can conclude therefore that heat loss is less efficient for Mars than Earth.

Our results on the present-day thermal state of Mars, and the corresponding construction of heat flow models and maps, are preliminary and a first step to characterizing the heat flow structure of the planet, and provide an understanding of the thermal history and dynamics of Mars. These results will also be helpful in evaluating specific landing zones for future missions to Mars; for example, contributing to the development of further studies on the distribution of groundwater or clathrates^56^,^57^, subsurface geological/biological production and transport of methane^58^, ice melting processes, localizing areas with higher thermal gradient, or other potential geological, geophysical and geochemical issues on the red planet. Also, our model can be improved with the constraints provided by additional observations, measurements and theoretical advances. The most immediate example is the InSight mission, which will provide heat flow measurements at a single location that according to our models are predicted to be 18 ± 1 mW m^−2^ at the proposed landing site at Elysium Planitia. The *in-situ* heat flow data can be implemented easily in this methodology, and will provide further anchoring for our model.

## Methods

### Crustal and mantle heat production

K and Th abundances on the surface have been estimated from measurements by the Gamma Ray Spectrometer (GRS) instrument on board the 2001 Mars Odyssey spacecraft^21^,^24^, and then corrected for volatile presence^18^, whereas U abundances are determined by assuming a Th/U ratio of 3.8 (e.g., ref. 18). As we use surface average HPEs abundances as representative for the whole crust (3652, 0.69 and 0.18 ppm for, respectively, K, Th and U), the corresponding average heat production of Mars is currently 4.86 × 10^−11^ W kg^−1^ (see also ref. 18). Concurrently, with a crustal density of 2900 kg m^−3^ (see below) this is equivalent to 0.1408 μW m^−3^, a contribution of ≈0.14 mW m^−2^ to the surface heat flow per each kilometer of crustal column. We assume HPEs homogeneously distributed in the crust, because the significant homogeneity of elemental abundances measure by Mars Odyssey GRS suggests that the martian crust is much less geochemically varied than the Earth’s crust^21^, which is consistent with a strong mixing by impact cratering^21^, and with the absence of large-scale crustal recycling, at least since the early part of the history of Mars^22^. Although there are some evidences suggesting that the crust of Mars could be stratified, with heat-producing elements concentrated in a layer thinner than the whole crust^12^,^59^, currently these evidences lack enough detail, and do not justify specifying whichever given crustal stratification structure in our modeling. Also, HPEs abundances in the mantle lithosphere are poorly constrained. Here we use HPEs mantle lithosphere abundances 0.1 times the average value for the martian crust (see ref. 8), which, for a mantle density of 3500 kg m^−3^ (see below), is equivalent to 0.0170 μW m^−3^, and translates to a contribution to the surface heat flow of ≈0.017 mW m^−2^ per each kilometer of mantle lithosphere column.

### Crustal thickness model

We use the relationship between global topography and gravity to model the crustal thickness (*T*_*c*_) of Mars following the potential theory procedure of Wieczorek and Phillips^25^. To constrain the thickness of the Martian crust, we assume (1) that the observed gravitational anomalies arises only from relief along the surface and crust–mantle interface, and (2) constant crustal and mantle densities to overcome the non-uniqueness associated with potential modeling. We use the spherical harmonic model MarsTopo2600 of the shape of Mars from Wieczorek^60^, and the gravitational potential model JGMRO_110 C from Konopliv *et al*.^61^. Topography data are useful only up to the resolution of gravity data, so both gravity and topography coefficients are truncated beyond degree and order 90 in our analysis given the dramatic decrease in spectral correlation that is observed between gravity and topography beyond this degree (e.g., ref. 32). It is further required either to assume a mean crustal thickness or to anchor the inverted crustal thickness to a given value at a specific location^60^. Here we assume a mean crustal thickness of 50 km (that satisfy the condition that the inverted crustal thickness is not negative anywhere on the planet). For crustal density we use 2900 kg m^−3^ for several reasons: this value is the usually used by works calculating crustal thickness from gravity and topography^60^,^62^ and the effective elastic thickness of the lithosphere (e.g., refs 4, 5, 13 and 26); a crustal density of 2900 ± 50 kg m^−3^ produces the best fit for the admittance/coherence signal of most of regions of Mars^4^,^5^; an upper limit of 3020 ± 70 kg m^−3^ has been calculated for the crustal density at the highlands of Mars^63^ from Geoid-to-topography ratios and Bouguer inversion. For the mantle lithosphere density, we assume 3500 kg m^−3^, a value widely used for Mars (e.g., refs 5, 8, 26 and 27). Under these assumptions, we first calculate the Bouguer gravity anomaly from surface topography and free air anomaly, and then calculate by downward continuation the shape of the crust–mantle interface necessary to minimize the difference between the observed and predicted Bouguer anomalies (for reviews see refs 25 and 60). In order to mitigate errors in downward continuing the Bouguer anomaly, we applied a minimum amplitude filter (see ref. 25) for the Moho relief at degree *l* = 50. Finally, we obtain the crustal thickness by subtracting the relief on the Moho from surface topography.

### Scaling of present-day surface heat flow

We scale heat flow differences across the martian surface due to crustal thickness and topographic differences in the planet using the expression *F*_*local*_ = *F*_*ref*_ + *H*_*c*_Δ*t *+* (H*_*c*_ − *H*_*m*_)(*Δb* − *Δt*), where *F*_*local*_ is the local heat flow for a given location on the surface of Mars, *F*_*ref*_ is a reference value used for anchoring the model, *H*_*c*_ and *H*_*m*_ are, respectively, the crustal and mantle heat production by volume unit, Δ*t* is the difference between the local elevation and the elevation at the reference location, and Δ*b* is the difference between the local crustal thickness and the crustal thickness in the reference location. The term *H*_*c*_*Δt* corresponds to the crustal contribution to the surface heat flow due to topographic differences across the surface; the term (*H*_*c*_ − *H*_*m*_)(*Δb* − *Δt*) corresponds to the effect from Moho topography and differences in heat production between crust and mantle. Surface topography and crustal thicknesses are derived, respectively, from Mars Orbiter Laser Altimeter (MOLA) topography^64^, and from our crustal thickness model described above. For reference location we take the North Polar Region (NPR), and we average crustal thickness and topography values in the region including 180°W to 180°E and 75°N to 90°N; in this case, for elevation determination it is necessary to subtract the average thickness of the north polar ice cap (2 km; refs 13, 14 and 65). The effective elastic thickness of the lithosphere (*T*_*e*_), which is a measure of the total strength of the lithosphere, can be converted to heat flow following the equivalent strength envelope procedure described by McNutt^66^. This methodology is based on the condition that the bending moment of the mechanical lithosphere must be equal to the bending moment of the equivalent elastic layer of thickness *T*_*e*_. The link between the mechanical structure and heat flow comes from the dependence of the ductile strength on temperature. Here we use the implementation of this methodology by Ruiz *et al*.^8^,^15^, which takes into account the strength contributions from the crust and the lithospheric mantle, and the effect of the polar cap above (but not included in) the lithosphere, in order to calculate an upper limit heat flow for the NPR. We calculate the temperature profile following the procedure described by Ruiz *et al*.^8^,^15^, and using crustal heat-producing elements (HPE) abundances based on Mars Odyssey GRS measurements (see above). We perform this calculation using a Cartesian geometry, because it is sufficient for deriving upper limit heat flows. Indeed, for a spherical geometry (which could be considered as appropriate due to the small radius of Mars): i) the temperature as a function of depth is somewhat increased, for a given heat flow, with respect to the Cartesian geometry, which in turn makes the temperature profile accordingly somewhat hotter; ii) increased temperatures in the lithosphere reduce the lithosphere strength; iii) because a fixed *T*_*e*_ implies necessarily a fixed lithosphere strength, in order to fit a given *T*_*e*_ derived from the observations, we must have a lower surface heat flow than in the Cartesian geometry. For *F*_*ref*_, we use a value of 17 mW m^−2^, which correspond to a heat flow calculated following this procedure, and taking for the NPR an effective elastic thickness of the lithosphere (*T*_*e*_) of 300 km (ref. 13), a crustal thickness of 33 km (after our crustal thickness model), an average elevation of −6 km, a thermal conductivity of 2 W m^−1^ K^−1^ for the crust, and a temperature-dependent thermal conductivity for the lithospheric mantle^8^. For comparative purposes, we have carried out in the same way an alternative heat flow model, based on scaling a *F*_*ref*_ of 23.5 mW m^−2^ deduced for the South Polar Region (180°W to 180°E and 80°S to 90°S) from *T*_*e*_ = 161 km^14^, and average crustal thickness and elevation of, respectively, 71 km and 1 km. We base our nominal model on the lithosphere strength in the NPR because *T*_*e*_ is better constrained than in the SPR (see ref. 15). For a complete explanation and discussion of the values used in the calculations see Ruiz *et al*.^8^,^15^. We also have performed a surface heat flow model from a *F*_*ref*_ of 17.5 mW m^−2^ at the NPR, based on an upper limit crustal thermal conductivity of 2.5 W m^−1^ K^−1^.

### Urey ratio calculations

We derive present-day Urey ratios by calculating the ratio between the total radioactive heat production obtained from the compositional model of Mars of Wänke and Dreibus^37^ and standard decay constants, and the average of present-day surface heat flow derived from our model. Thus, Urey ratio is given by *Ur* = *H*_M_*M*_*mars*_/(4π*R*_*mars*_^2^*F*), where *H*_M_ is the mean heat production rate by mass unit, *M*_*mars*_ and *R*_*mars*_ are, respectively, the mass and the mean radius of Mars, and *F* is the average surface heat flows.

## Additional Information

**How to cite this article**: Parro, L. M. *et al*. Present-day heat flow model of Mars. *Sci. Rep.* **7**, 45629; doi: 10.1038/srep45629 (2017).

**Publisher's note:** Springer Nature remains neutral with regard to jurisdictional claims in published maps and institutional affiliations.

## References

[b1] Ruiz J. The early heat loss evolution of Mars and their implications for internal and environmental history. Sci. Rep. 4, 4338, doi: 10.1038/srep04338 (2014). 24614056 PMC3949296

[b2] Spohn T. . InSight: Measuring the Martian Heat Flow using the Heat Flow and Physical Properties Package (HP3). Lunar Planet. Sci. Conf. 43, 1445 (2012).

[b3] Solomon S. C. & Head J. W. Heterogeneities in the thickness of the elastic lithosphere of Mars: Constraints on heat flow and internal dynamics. J. Geophys. Res. 95, 11073–11083 (1990).

[b4] McGovern P. J. . Localized gravity/topography admittance and correlation spectra on Mars: Implications for regional and global evolution. J. Geophys. Res. 107, 5136, doi: 10.1029/2002JE001854 (2002).

[b5] McGovern P. J. . Correction to Localized gravity/topography admittance and correlation spectra on Mars: implications for regional and global evolution. J. Geophys. Res. 109, E07007, doi: 10.1029/2004JE002286 (2004).

[b6] Grott M., Hauber E., Werner S. C., Kronberg P. & Neukum G. High heat flux on ancientMars: Evidence from rift flank uplift at Coracis Fossae. Geophys. Res. Lett. 32, L21201, doi: 10.1029/2005GL023894 (2005).

[b7] Ruiz J., McGovern P. J. & Tejero R. The early thermal and magnetic state of the cratered highlands of Mars. Earth Planet. Sci. Lett. 241, 2–10 (2006).

[b8] Ruiz J. . The thermal evolution of Mars as constrained by paleo-heat flows. Icarus 215, 508–517 (2011).

[b9] Schultz R. A. & Watters T. R. Forward mechanical modeling of the Amenthes Rupes thrust fault on Mars. Geophys. Res. Lett. 28, 4659–4662 (2001).

[b10] Grott M., Hauber E., Werner S. C., Kronberg P. & Neukum G. Mechanical modelling of thrust faults in the Thaumasia region, Mars, and implications for the Noachian heat flux. Icarus 186, 517–526 (2007).

[b11] Ruiz J. . Ancient heat flow, crustal thickness, and lithospheric mantle rheology in the Amenthes region, Mars. Earth and Planetary Science Letters 270, 1–12 (2008).

[b12] Ruiz J. . Ancient heat flows and crustal thickness at Warrego rise, Thaumasia Highlands, Mars: Implications for a stratified crust. Icarus 203, 47–57 (2009).

[b13] Phillips R. J. . Mars north polar deposits: stratigraphy, age, and geodynamical response. Science 320, 1182–1185 (2008). 18483402 10.1126/science.1157546

[b14] Wieczorek M. A. Constraints on the composition of the martian south polar cap from gravity and topography. Icarus 196, 506–517 (2008).

[b15] Ruiz J., López V. & Dohm J. M. The present-day thermal state of Mars. Icarus 207, 631–637 (2010).

[b16] Laskar J., Levrard B. & Mustard J. F. Orbital forcing of the martian polar layered deposits. Nature 419, 375–377 (2002). 12353029 10.1038/nature01066

[b17] Grott M. & Breuer D. On the spatial variability of the Martian elastic lithosphere thickness: Evidence for Mantle plumes? J. Geophys. Res. 115, E03005, doi: 10.1029/2009JE003456 (2010).

[b18] Hahn B. C., McLennan S. M. & Klein E. C. Martian surface heat production and crustal heat flow from Mars Odyssey Gamma-Ray spectrometry. Geophys. Res. Lett. 38, L14203, doi: 10.1029/2011GL047435 (2011).

[b19] Parro L. M., Jiménez-Díaz A. & Ruiz J. Current thermal state of Mars from scaled models of surface heat flow. EPSC Abstracts 10, EPSC2015-499 (2015).

[b20] Plesa A.–C., Grott M., Tosi N., Breuer D & Spohn T. Present-day heat flux variations across the surface of Mars. Lunar Planet. Sci. Conf. 47, 1931 (2016).

[b21] Taylor G. J. . Variations in K/Th on Mars. J. Geophys. Res., 111, E03S06, doi: 10.1029/2006JE002676 (2006a).

[b22] Frey H. V. Impact constraints on, and a chronology for, major events in early Mars history. J. Geophys. Res. 111, E08S91, doi: 10.1029/2005JE002449 (2006).

[b23] Taylor G. J. . Bulk composition and early differentiation of Mars. J. Geophys. Res. 111, E03S10, doi: 10.1029/2005JE002645 (2006b).

[b24] Boynton W. V. . Concentration of H, Si, Cl, K, Fe, and Th in the low and mid latitude regions of Mars. J. Geophys. Res. 112, E12S99, doi: 10.1029/2007JE002887 (2007).

[b25] Wieczorek M. A. & Phillips R. J. Potential anomalies on a sphere: applications to the thickness of the lunar crust. J. Geophys. Res. 103, 1715–1724, doi: 10.1029/97JE03136 (1998).

[b26] Zuber M. T. . Internal structure and early thermal evolution of Mars from Mars Global Surveyor. Science 287, 1788–1793 (2000). 10710301 10.1126/science.287.5459.1788

[b27] Neumann G. A., Zuber M. T., Wieczorek M. A., McGovern P. J., Lemoine F. G. & Smith D. E. Crustal structure of Mars from gravity and topography. J. Geophys. Res. 109, E08002, doi: 10.1029/2004JE002262 (2004).

[b28] Neumann G. A., Lemoine F. G., Smith D. E. & Zuber M. T. Marscrust3-A crustal thickness inversion from recent MRO gravity solutions. Lunar Planet. Sci. Conf. 39, 2167 (2008).

[b29] Wieczorek M. A. & Zuber M. T. Thickness of the Martian crust: improved constraints from geoid-to-topography ratios. J. Geophys. Res. 109, E01009, doi: 10.1029/2003JE002153 (2004).

[b30] Carter J. & Poulet F. Ancient plutonic processes on Mars inferred from the detection of possible anorthositic terrains. Nat. Geosci. 6, 1008–1012, doi: 10.1038/ngeo1995 (2013).

[b31] Wray J. J. . Prolonged magmatic activity on Mars inferred from the de- tection of felsic rocks. Nat. Geosci. 6, 1013–1017 (2013).

[b32] Baratoux D. . Petrological constraints on the density of the Martian crust. J. Geophys. Res. Planets 119, 1707–1727 (2014).

[b33] Sautter V. . *In situ* evidence for continental crust on early Mars. Nat. Geosci. 8, 605–609 (2015).

[b34] Rogers A. D. & Nekvasil H. Feldspathic rocks on Mars: compositional con- straints from infrared spectroscopy and possible formation mechanisms. Geo- phys. Res. Lett. 42, 2619–2626 (2015).

[b35] Beardsmore G. R & Cull J. P. Crustal Heat Flow. A Guide to Measurement and Modelling, Cambridge University Press, Cambridge (2001).

[b36] Jaupart C., Mareschal J.-C. & Iarotsky L. Radiogenic heat production in the continental crust. Lithos. 262, 398–427 (2016).

[b37] Wänke H. & Dreibus G. Chemical composition and accretion history of terrestrial planets. Philos. Trans. Roy. Soc. London: Ser. A 325, 545–557 (1988).

[b38] Plesa A.-C., Tosi N., Grott M. & Breuer D. Thermal evolution and Urey ratio of Mars. J. Geophys. Res. Planets 120, 995–1010, doi: 10.1002/2014JE004748 (2015).

[b39] Parmentier E. M. & Zuber M. T. Early evolution of Mars with mantle compositional stratification or hydrothermal crustal cooling. J. Geophys. Res. 112, E02007, doi: 10.1029/2005JE002626 (2007).

[b40] Kiefer W. S. & Li Q. Mantle convection controls the observed lateral variations in lithospheric thickness on present-day Mars. Geophys. Res. Lett. 36, L18203, doi: 10.1029/2009GL039827 (2009).

[b41] Burr D. M., McEwen A. S. & Sakimoto S. E. H. Recent aqueous floods from the Cerberus Fossae, Mars. Geophys. Res. Lett. 29, 1013, doi: 10.1029/2001GL013345 (2002).

[b42] Zhong S. J. & Roberts J. H. On the support of the Tharsis Rise on Mars. Earth Planet. Sci. Lett. 214, 1–9 (2003).

[b43] Roberts J. H. & Zhong S. J. Plume-induced topography and geoid anomalies and their implications for the Tharsis rise on Mars. J. Geophys. Res. 109, E03009, doi: 10.1029/2003JE002226 (2004).

[b44] Plesa A.-C. & Breuer D. Partial melting in one-plate planets: Implications for thermo-chemical and atmospheric evolution. Planet. Space Sci. 98, 50–65, doi: 10.1016/j.pss.2013.10.007 (2014).

[b45] Hauck S. A. II & Phillips R. J. Thermal and crustal evolution of Mars. J. Geophys. Res. 107(E7), doi: 10.1029/2001JE001801 (2002).

[b46] Williams J.-P. & Nimmo F. Thermal evolution of the martian core: Implications for an early dynamo. Geology 32, 97–100 (2004).

[b47] Fraeman A. A. & Korenaga J. The influence of mantle melting on the evolution of Mars. Icarus 210, 43–57 (2010).

[b48] Morschhauser A., Grott M. & Breuer D. Crustal recycling, mantle dehydration, and the thermal evolution of Mars. Icarus 212, 541–558 (2011).

[b49] Baratoux D., Toplis M. J., Monnereau M. & Gasnault O. Thermal history of Mars inferred from orbital geochemistry of volcanic provinces. Nature 472, 338–341 (2011). 21471967 10.1038/nature09903

[b50] Yoder C. F., Konopliv A. S., Yuan D. N., Standish E. M. & Folkner W. M. Fluid core size of Mars from detection of the solar core. Science 300, 299–303 (2003). 12624177 10.1126/science.1079645

[b51] Hauber E., Broz P., Jagert F., Jodlowski F. & Platz T. Very recent and wide-spread basaltic volcanism on Mars. Geophys. Res. Lett. 38, L10201, doi: 10.1029/2011GL047310 (2011).

[b52] Nahm A. L. & Schultz R. A. Magnitude of global contraction on Mars from analysis of surface faults: implications for Martian thermal history. Icarus 211, 389–400 (2011).

[b53] Musselwhite D. S., Dalton H. A., Kiefer W. S. & Treiman A. H. Experimental petrology of the basaltic shergottite Yamato-980459: implications for the thermal structure of the Martian mantle. Meteor. Planet. Sci. 41, 1271–1290 (2006).

[b54] Collinet M., Médard E., Devouard B. & Peslier A. Constraints on the parental melts of enriched shergottites from image analysis and high pressure experiments. Lunar Planet. Sci. Conf. 43, 2269 (2012).

[b55] Filiberto J. & Dasgupta R. Constraints on the depth and thermal vigor of melting in the Martian mantle. J. Geophys. Res. Planets 120, 109–122 (2015).

[b56] Gloesener E., Karatekin O. & Dehant V. Martian methane and link with clathrates in the crust of Mars. EGU, 15, 5174 (2013).

[b57] Jaupart C., Labrosse S. & Mareschal J.-C. Temperatures, heat and energy in the mantle of the Earth, in Treatise on Geophysics. Vol 7 (eds. Schubert G.), 253–304 (Elsevier, 2007).

[b58] Stevens A. H., Patel M. R. & Lewis S. R. Numerical modelling of the transport of trace gases including methane in the subsurface of Mars. Icarus 250, 587–594 (2015).

[b59] Egea-Gonzalez I. . Thrust fault modeling and Late-Noachian lithospheric structure of the circum-Hellas region, Mars. Icarus 288, 53–68 (2017).

[b60] Wieczorek M. A. Gravity and Topography of the Terrestrial Planets. Treatise on Geophysics 2nd edition vol. 10, 153–193 (2015).

[b61] Konopliv A. S. . Mars high resolution gravity fields from MRO, Mars seasonal gravity, and other dynamical parameters. Icarus 211, 401–428 (2011).

[b62] Genova A. . Seasonal and static gravity field of Mars from MGS, Mars Odyssey and MRO radio science. Icarus 272, 228–245 (2016).

[b63] Pauer M. & Breuer D. Constraints on the maximum crustal density from gravity topography modeling: Applications to the southern highlands of Mars. Earth Planet. Sci. Lett. 276, 253–261 (2008).

[b64] Smith D. E. . Mars Orbiter Laser Altimeter: Experiment summary after the first year of global mapping of Mars. J. Geophys. Res. 106, 23689–23722, doi: 10.1029/2000JE00136 (2001).

[b65] Plaut J. J. . Subsurface radar sounding of the south polar layered deposits of Mars. Science 316, 92–95 (2007). 17363628 10.1126/science.1139672

[b66] McNutt M. K. Lithospheric flexure and thermal anomalies. J. Geophys. Res. 89, 11180–11194 (1984).

